# Characterization of cells recovered from the xenotransplanted NG97 human-derived glioma cell line subcultured in a long-term *in vitro*

**DOI:** 10.1186/1471-2407-8-291

**Published:** 2008-10-08

**Authors:** Camila ML Machado, Rafael Y Ikemori, Tatiana Q Zorzeto, Ana CMA Nogueira, Suse DS Barbosa, Wilson Savino, André A Schenka, José Vassallo, Juliana K Heinrich, Fátima Boetcher-Luiz, Liana Verinaud

**Affiliations:** 1 Department of Microbiology and Immunology, Institute of Biology, UNICAMP – Campinas, São Paulo, Brazil; 2 Laboratory of Investigative and Molecular Pathology-CIPED, Faculty of Medical Sciences, UNICAMP – Campinas, São Paulo, Brazil; 3 Clinical Specialized Laboratories, Centre of Integral Service to the Health of the Woman-CAISM, UNICAMP – Campinas, São Paulo, Brazil; 4 Department of Obstetrics and Gynecology, Faculty of Medical Sciences, UNICAMP – Campinas, São Paulo, Brazil; 5 Department of Immunology, National Institute of Quality Control and Health, INCQS, FIOCRUZ – Rio de Janeiro, Rio de Janeiro, Brazil; 6 Laboratory on Thymus Research – FIOCRUZ – Rio de Janeiro, Rio de Janeiro, Brazil

## Abstract

**Background:**

In order to elucidate tumoral progression and drug resistance, cultured cell lines are valuable tools applied on tumor related assays provided they are well established and characterized. Our laboratory settled the NG97 cell line derived from a human astrocytoma grade III, which started to develop and express important phenotypical characteristics of an astrocytoma grade IV after injection in the flank of nude mice. Astrocytomas are extremely aggressive malignancies of the Central Nervous System (CNS) and account for 46% of all primary malignant brain tumors. Progression to worse prognosis occurs in 85% of the cases possibly due to changes in cell tumor microenvironment and through biological pathways that are still unclear.

**Methods:**

This work focused on characterizing the NG97 cell line specifically after being recovered from the xenotransplant, who maintained their undifferentiated characteristics along the following 60^th ^passages in *vitro. *These cells were subcultivated to evaluate the possible contribution of these undifferentiated characteristics to the malignant progression phenotype. These characteristics were the expression of molecules involved in the processes of migration, dedifferentiation and chromosomal instability.

**Results:**

Results showed that NG97(ht) had an decrease in doubling time through sub cultivation, which was characterized by a converse modulation between the expression of glial fibrillary acidic protein (GFAP) and vimentin. In addition, β1 integrins were present in intermediate levels while α5 integrins had a high expression profile as well as fibronectin and laminin.

Cytogenetic analysis of NG97(ht) revealed several chromosomal abnormalities, 89% of the cells showed to be hyperdiploid and the modal number was assigned to be 63. Several acrocentric chromosomes were visualized and at least 30 figures were attributed to be murine. These findings suggest a possible fusion between the original NG97 cells with stromal murine cells in the xenotransplant.

**Conclusion:**

In this study the NG97(ht) cells were characterized to embryonic recovery patterns of intermediate filaments, adhesion molecules expression, chromosomal imbalances and murine chromosomes. In the latter case, these presumably chromosomes were originated as fusions between murine stroma cells and NG97 cell lineage in the xenotransplant. Our results emphasize important queries about astrocytomas tumor progression.

## Background

Astrocytomas are highly aggressive tumors that account for around 46% of all the primary malignancies of the Central Nervous System (CNS), demonstrate poor prognosis and statistics show a 5-year survival ranging from 22% for astrocytomas-grade III to only 2% for astrocytomas-grade IV after diagnosis [1]. The treatment is surgical excision followed by adjuvant chemotherapy [2] and radiotherapy; however, many patients exhibit recurrences due to intrinsic drug resistance within 2 years following the removal of the tumoral mass, leading to death [3].

A better understanding of tumor dynamics and progression pathways will improve both diagnosis and therapeutics. For this regard, many laboratories have established cell lines from tumors [4-7]. In the same way, the NG97 glioma cell line was recently established in our laboratory after the removal of a tumor mass from a patient who had been diagnosed with an astrocytoma grade III [8]. The subcutaneous inoculation of NG97 cells in the flank of athymic mice (nu/nu) resulted in the development of solid tumor masses, demonstrating its tumorigenicity [8]. When the tumor mass was excised and examined, a spontaneous tumor progression was confirmed by the presence of prominent vascularity, presence of pseudopalisading cells and increase of GFAP which were compatible with a grade IV astrocytoma or glioblastoma multiforme [9]. Cells from the tumor mass were then processed and cultivated *in vitro *as an adherent monolayer and had the same morphological characteristics of the original culture, before the xenotransplant [8].

Many authors report the tumor progression phenotype as a result of expression of dedifferentiated characteristics of the cells. During the embryonic development of the CNS, astrocytes hypothetically are originated from progenitors that solely express vimentin as a cytoskeleton filament [10,11]. These cells have a migratory pattern and before they migrate to the glia radial, they express vimentin and GFAP during cell maturation period [12]. By the end of this process, mature cells express mainly GFAP [13] as a cytoskeleton protein. In the adult brain, most of glial cells express GFAP and this expression can be modified in the course of many diseases such as Alzheimer's when they become positive or even negative as in astrocytomas [14]. For these tumors, a GFAP and vimentin proteomics modulatory pattern was described in patients who progressed from grade III to IV [15,16].

The migration pattern presented by glioma cells can be associated to the progenitor and embryonic CNS cell migration [17]. The transformed cells that reach a malignant progression, acquire the ability to migrate through tissues in the tumor microenvironment, consequently resulting in tumor mass growth. This infiltration ability is driven by a set of molecules called integrins and their receptors in the extracellular matrix [18]. In gliomas, the most representatives of this group are a quite a few forms of α and β1 integrins, laminin and fibronectin. [19-21].

Considering the tumor progression, since 70's decade, there is a consensus about the genetic instability resulting in clones that would become more aggressive after successive mitotic divisions [22]. About five decades earlier, Boveri (1929) [23] observed that sea urchin eggs experimentally fertilized with two (rather than one) sets of spermatozoa underwent abnormal mitosis and proposed that the deregulated growth of cancer cells might also be a result of chromosomal imbalance. These findings have led some researchers to postulate that *in vivo *cell fusion in cancer [24] would not only be responsible for chromosomal abnormalities, but also for the acquisition of the malignant phenotype and metastasis promotion [25,26].

In this study, we investigated the expression of astrocytic molecules of embryonic origin, adhesion and deadhesion molecules and the cytogenetic patterns of the NG97(ht); and its characterization may clarify the tumor spontaneous progression into astrocytoma.

## Methods

### Cell culture

The NG97(ht) xenotransplanted cultures were derived from the NG97 cell line. It was firstly described in 2001 [4] and obtained through the excision, cutting and enzymatic digestion of the tumor mass of the xenotransplant and *in vitro *cultivation of the small pieces, according to the explant technique. Subcultures were carried out in D-valine-containing Eagle's minimal Essential Medium (GIBCO) supplemented with 2 mmol/L L-glutamine, 10 mmol/L HEPES, 100 U/mL penicillin, 100 μg/mL streptomycin, and 10% heat-inactivated fetal calf serum (Nutricell, Campinas-SP-Brazil) and stocked as described before [4]. After being defrost and adapted to the cultivation for five passages, cells were cultivated by more than a hundred passages in RPMI-1640, supplemented with 13% heat-inactivated fetal calf serum (Nutricell, Campinas, SP, Brazil) and 25 μg/mL streptomycin. Cultures were maintained in a humidified atmosphere at 37°C and 5% CO_2_. The medium was changed after intervals of 24 h when the culture almost reached confluence. Sub culturing was performed after treatment with trypsin and versene (Adolfo Lutz, São Paulo, SP, Brazil). In this moment, the NG97(ht) cell line was underwent about 120 passages.

### Subcultivations growths curves and cells passages doubling time

The amount of 1 × 10^4 ^cells of NG97 cells was cultivated in triplicates on 24-well plate for 11 days to determine the growth curve. After processing the cultures with the routine TVS solution treatment (tripsin-versene solution), aliquots of the cell suspension obtained was diluted daily in 1% trypan blue in RPMI medium and counted in a Newbauer chamber.

The number of cells duplications in culture was calculated according to the formula: (1.1) N = N_0 _× 2^n^, where "N" is the final number of cells after 11 days of culture, N_0 _the number at the beginning of the exponential growth phase of culture and "n" the number of duplications in the amount of cells.

For the determination of the doubling time (T), the following formulas were used: (1.2) *g *= t - t_0_, where "t" represents the final time in hours when "n" was determined (as described and determined above in (1.1) equation) and "t_0_"the initial time when N_0 _was studied. Each final cell doubling time was obtained by the mathematical formula: T = g/n [27].

### Western blotting reaction

total protein extraction was carried out after cells being homogenized in 1% Triton X-100, 50 mM PB pH 7.4, 1 mM sodium pirophosphate, 1 mM sodium fluoride, 5 mM EDTA, 1 mM sodium vanadate, 1% protease inhibitor cocktail (P8340 Sigma), 7 M Urea, 2 M Tiourea. Sample homogenization was carried out at 4°C using a Politron 20 s generator (Brinkmann) set at maximum speed for 30 seconds. To remove insoluble materials, centrifugation (12,000 g centrifugation, 4°C for 15 minutes) was performed. Protein concentration was determined using the Bradford method [28]. Total protein extracts from each cell sample were eletrophoretically separated in SDS-PAGE and electro blotted to a nitrocellulose membrane according to standard procedures [29]. Membranes were blocked with PBS-tween^® ^containing 5% non-fat dry milk and than incubated with an anti-GFAP (polyclonal antibody, rabbit anti-bovine GFAP) (cat. no. Z0334 from DakoCytomation, California, USA) and anti vimentin (mouse monoclonal antibody, Vim clone 3B4) (DakoCytomation, California, USA) diluted (1:1,000) in PBS-tween containing 3% BSA for 12 h at 4°C. Membranes were washed with PBS-tween^® ^and incubated with HRP labeled secondary antibody (Zymed, 1:10,000). Reactive bands were detected with the SuperSignal West Pico chemiluminescent kit (Pierce).

### Immunocytochemistry

NG97(ht) cells from the 19^th ^to 83^rd ^passages were grown on 13 mm sterile rounded cover slips which were rinsed three times in phosphate saline buffer (PBS), pH 7,4 at RT, and treated with 3% H_2_O_2 _in methanol to suppress endogenous peroxidase activity. After being washed in PBS, slides were treated with 1% normal mouse serum at RT for 1 hour. The slides were washed again in PBS, and then incubated overnight at 4°C with the following antibodies for GFAP (anti-rabbit polyclonal anti-GFAP, cat. no. Z0334 from DakoCytomation, California, USA) and anti-vimentin (mouse monoclonal antibody, clone V9, DakoCytomation, California, USA). All incubations were carried out in a darkened, humidified chamber at RT. After being washed in PBS, the slides were incubated with labeled polymer horseradish peroxidase anti-mouse/anti-rabbit (EnVysion plus^® ^System, DakoCytomation; Carpinteria, CA, USA) for 1 hour at RT. Peroxidase enzyme activity was visualized by DAB solution (DAKO liquid DAB substrate-chromogen solution). To stop the reaction distilled water was applied. The slides were then weakly counter-stained with Harris' Haematoxylin, dehydrated in an ethanol series and mounted in Permount^® ^medium. The images were captured with a Nikon microscope connected to an image acquiring system (Leyca^® ^system). Positive cells were counted using specific software (Pro-plus 4.3 software^®^). For each batch positive and negative controls were used.

### Flow Cytometry

Cells from the 22^nd^, 54^th ^and 92^nd ^passages were harvested from culture flasks, washed and re-suspended in cold PBS supplemented with 0.5% BSA. Then, cells were incubated for 30 minutes at RT in the dark with monoclonal mouse antibodies such as: anti-β1-PECy5, anti-α4-PE, anti-α5-PE, anti-α6-PE and isotypes controls such as mouse IgG-PECy5 and IgG-PE (BD biosciences^®^-Pharmingen, Mountain View, CA, USA). The expression of these surface molecules were analyzed by flow cytometry (FACSCalibur^®^, BD Biosciences, Mountain View, CA, USA) and quantified by WinMdi^® ^shareware software. The cloned human thymic epithelial cell line described by Fernandez and co-workers [30] was included as a positive control for the flow cytometry reaction.

### Immunofluorescence

the slides containing NG97(ht) were rinsed in PBS and incubated for 1 h at 37°C with mouse anti-human fibronectin and anti-human laminin antibodies (BD Biosciences, Mountain View, CA, USA). For detection, slides were incubated for 2 hours at RT with FITC-conjugated rabbit anti-mouse serum (Santa Cruz Biotechnology Inc., California, CA, and USA). Slides were mounted using the ProLong Antifade Kit (Molecular Probes, Eugene, OR, USA) according to the manufacturer's instructions, and immunoreactions were viewed using an Olympus fluorescent microscopic coupled to a Kodak camera. Negative controls in which normal mouse immunoglobulin was substituted by the primary antibody were included as well as positive controls with the cloned human thymic epithelial cell line described elsewhere [30].

### Cytogenetics

The analysis of the 20^th ^passage was obtained after routine cell culture for karyotype. Briefly metaphase spreads were obtained after 6-hour incubation with 100 μl of Karyomax 10 μg/ml (GIBCO). GTG and C-banding were assessed through standard banding protocols. Image analysis and acquisition were performed in a Zeiss Axioplan microscope 2 ^® ^equipped with the BandView software (Applied Spectral Imaging). The results of the several analyses were compaired at the same passage or, when it is impossible, in a representing of a near passage for analysis.

### Statistical analysis

A linear regression was calculated to verify a relation between the duplication time and the progression of cell passages in culture, with the software SYSTAT 10, 2 for Windows (SYSTAT Software Incorporation, 2002). For the validation of the described model, the analysis of residues was also assessed.

### Results

#### Cell Growth Kinetics

The NG97(ht) cell growth was observed in the 19^th^, 24^th^, 32^nd^, 43^rd^, 59^th^, 65^th ^and 73^rd ^cell passages. This study of several passages in culture improves the knowledge about the maintenance or lack of all features observed in these cells from *in vivo *tumoral mass to cells in long-term subcultivation *in vitro*.

**Figure 1:**
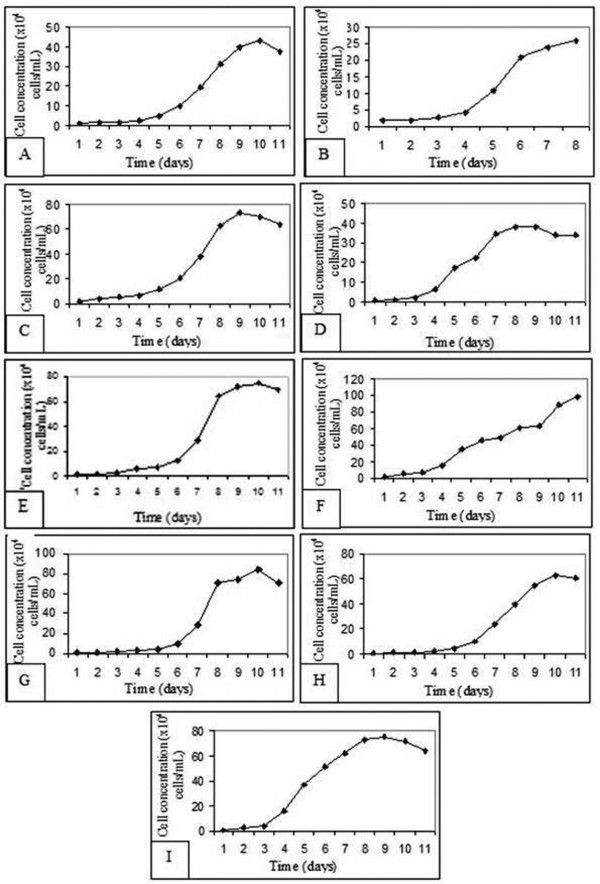
Representative cell growth graphics of different NG97(ht) cells passage through subcultivation: 19^th ^(A), 24^th ^(B), 32^nd ^(C), 37^th ^(D), 43^rd ^(E), 51^st ^(F), 59^th ^(G), 65^th ^(H) e 73 ^rd ^(I).

**Figure 2:**
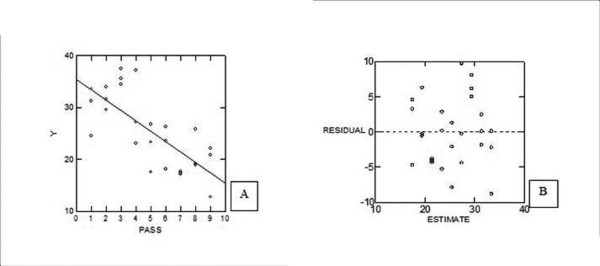
**Statistical analysis to validate the significance of the NG97(ht) doubling time decrease.** The simple linear regression was used and show the correlation between cell doubling time (plotted in "Y" axis) and the cell passages at subculture, which was represented by ordinal values (plotted in "X" axis). The equation that describes the linear regression mathematical model was y = 35.379-1.994x (A). (B) This graphic represents the residual analysis to validate the mathematic model (R^2 ^= 715,415; F_1,25 _= 30,597 and P << 0,001).

All curves revealed a typical pattern of cell kinetics and growth including four distinct phases: an initial phase which is represented by slight cells divisions (i), followed by an exponential growth (ii) and a stationary phase (iii). An end phase represented by a growth decline as well as cell death (iv) is shown in figure 1. The doubling time was calculated for each cell passage (Table 1) assayed and the statistical analysis showed a negative linear correlation between the NG97(ht) doubling time through the subcultivation passages, which demonstrates a significant doubling time decrease (fig. 2A). This linear model regression was validated by residual analysis (R^2 ^= 715,415; F_1,25 _= 30,597 e P << 0,001) as represented in fig. 2B.

**Table 1:** Increase of cell proliferation by doubling culture time decreased calculated to NG97(ht) trough cell subcultivation

Cell passages	Doubling time (hours)
19	30
24	32
32	36
37	29
43	23
51	23
59	17
65	21
73	19

* Approximated values.

### GFAP and vimentin patterns

In order to assess the ontogenic protein patterns and their stability through cell subcultivation: early (until 50th), middle (from 51^st ^to 79^th^) and later (after 80^th^) NG97(ht) cell passages were analyzed.

**Figure 3:**
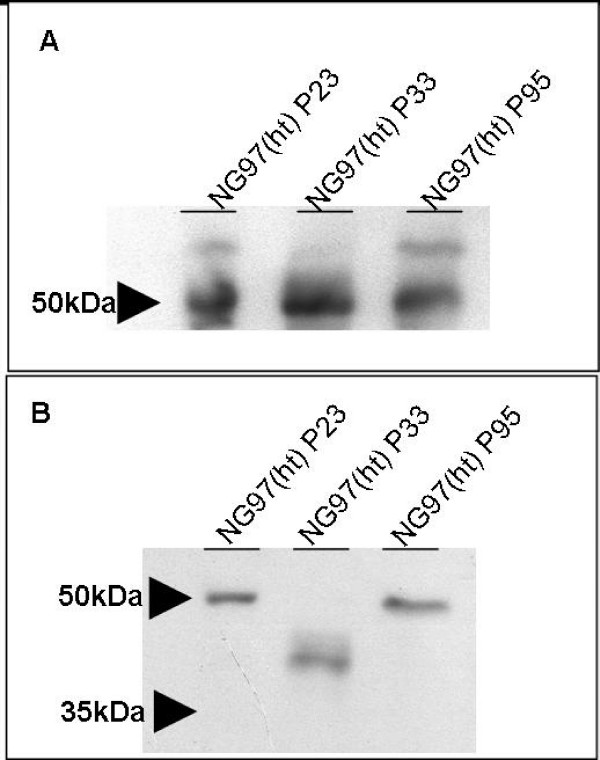
**Western Blot of NG97(ht) from different cell passages subcultivation to (A) GFAP and (B) vimentin, which contained approximately 5 μg and 30 μg of protein cytoplasm fraction, respectively.** Lines 1, 2 and 3 show 23^rd^, 33^rd ^e 95^th ^cell passages, respectively. (A) Note the GFAP basal linear conformational protein expression banded at approximated 50 kDa molecular weight in all passages. (B) Note the vimentin linear conformational basal expression banded at approximated 50 kDa molecular weight at earlier and later passages, though a 41,8 kDa molecular weight banded the 33^rd ^cell passage resulted from a partial vimentin degradation by the protein sonication extraction procedure.*(Positive and negative controls were assayed but were omitted here).*

Western Blot (WB) reactions showed that cells expressed basal amounts of GFAP (fig. 3A) and Vimentin (fig. 3B) cytoskeleton proteins in the cytoplasm fraction containing 5 μg and 30 μg amounts of proteins, respectively. In particular, another molecular weight (approximately 41,8 kDa) for vimentin was also registered from protein degradation during 33^rd ^cell passage protein extraction by sonication.

**Figure 4:**
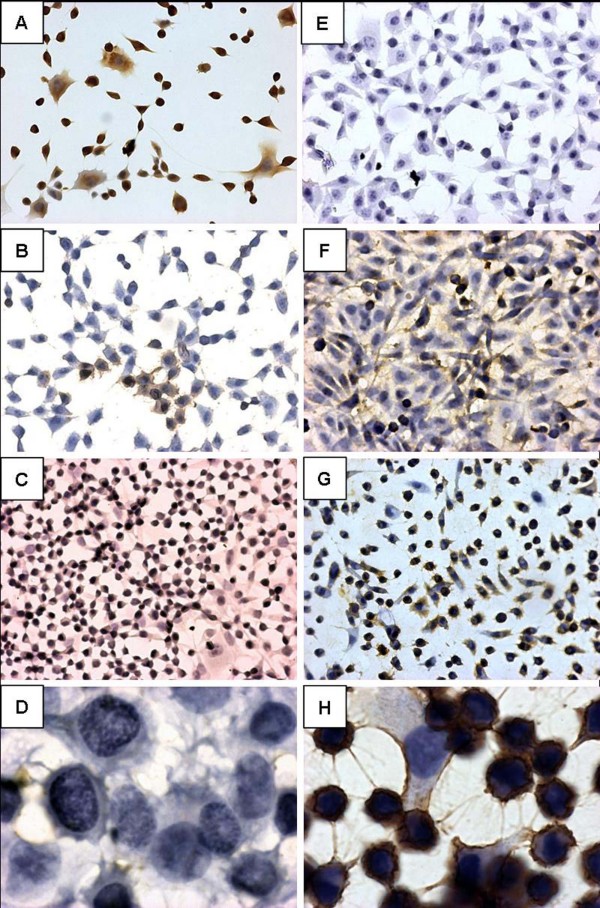
**GFAP and vimentin immunocytochemistry through cell subcultivation.** Notice the negative modulatory expression pattern to GFAP (A – D) concomitant to the positive modulatory pattern to vimentin (E-H) in NG97(ht). The GFAP appears intensely marked in 100% of the 19^th ^cell passage cytoplasm (A), being presented in approximately 85% of the 30^th ^(B) and, finally, totally absent in 51^st ^(C) and 80^th ^(D) cell passages. Nonetheless, the vimentin were absent at 21^st ^(E) cell passage, presented in weakly areas in the 40^th ^(F) and in 54% of the 59^th ^(G) cell passage and, finally, markedly positive at 83^rd ^(H) cell passage *(Original magnification: A – C and E – G = 200× and D and H = 1000×) (Positive and negative controls were assayed but were not displayed here).*

This observed GFAP detection did not represent the functional molecular polymerized expression. These GFAP and vimentin cytoskeletons conformational arrangements were demonstrated by immunocytochemistry and suggested a modulation pattern through cell subcultivation. An intense cytoplasmatic staining for GFAP was observed in 100% of cells in the 19^th ^cell passage (fig. 4A). The same pattern was present in approximately 85% of the NG97(ht) cells in the 30^th ^(fig. 4B) and, finally, totally absent in 51^st ^(fig. 4C) and 80^th ^(fig. 4D) cell passages. Nevertheless, it was shown an absence of staining for cytoplasmatic vimentin in the 21^st ^cell passage (fig. 4E), followed by weakly stained areas in the 40^th ^NG97(ht) cell passage (fig. 4F). Approximately 54% of NG97(ht) cells in the 59^th ^passage (fig. 4G) and, finally, a markedly positive staining in the 83^rd ^cell passage (fig. 4H).

### Expression of integrins and their receptors

Flow cytometry was applied on passages 22^nd^, 54^th ^and 92^nd ^for the assay of β1, α4, α5 and α6 subunits (Table 2).

**Table 2:** Absence of α4 and α6 integrin subunits.

	NG97(ht)				
Markers	early	middle	Later	average	SD

β1 (CD29)	49,0	58,47	50,70	52,72	5,04

α4 (CD49d)	0,0	0,0	0,0	0,0	0,0

α5 (CD49e)	98,03	98,72	98,21	98,32	0,35

α6 (CD49f)	0,0	0,0	0,0	0,0	0,0

Intermediate β1 and high α5 integrin expression levels in: earlier (22^nd^), middle (54^th^) and later (92^nd^) NG97(ht) cell passages by flow cytometry. The results showed the percentage of positive cells, the average of positivity for each marker and the standard deviation (SD).

*Representative values of 10^4 ^cells NG97(ht) analyzed. (Positive controls with the cloned human thymic epithelial cell line was assayed but was not displayed here).

The table 2 shows a low average expression of the β1 chain (52,72%) through cell subcultivation, when compared to the α5 subunit that presented an average of 98,32% of positive cells through sulbcultivation. The α4 and α6 integrins were not detected. The positive control, a cloned human thymic epithelial cell line established by Fernandez and co-workers [30], showed a 95–99% range of expression through flow cytometry for β1, α4, α5 and α6 subunits (not shown).

**Figure 5:**
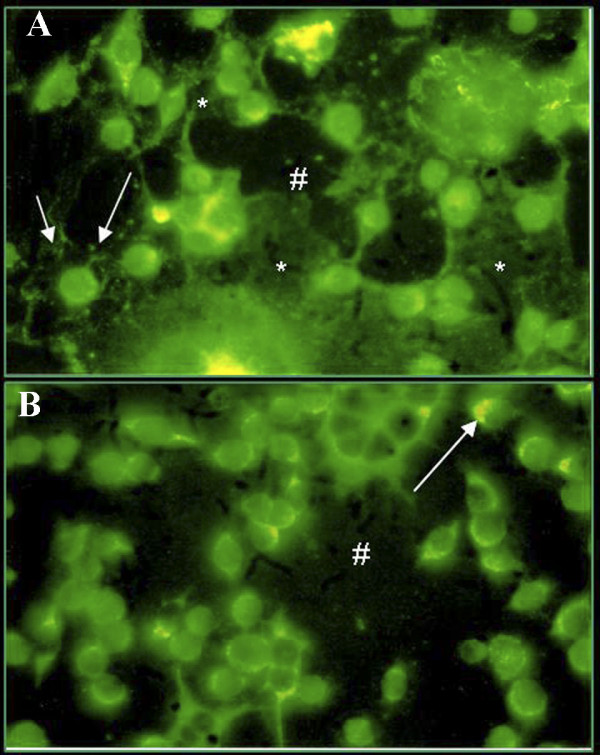
Immunofluorescence in the NG97(ht) 20^th ^cell passage for (A) fibronectin and (B) laminin expression which were represented by a diffusely cytoplasmatic pattern, hot spot areas (#) and some fibrils (*) deposition in the ECM and increase of polymerization in the lamelipodia cytoplasmatic extensions (arrow) (original magnification: 400×).*(Positive controls with cells of cloned human thymic epithelial lineage were assayed; however the data are not displayed here).*

Fibronectin and laminin staining was performed on fixed cells, which may illustrate the intracellular and extracellular presence of proteins on living cells. A punctate fibronectin and laminin immunoreactivity mainly located in the perinuclear area was observed (fig. 5A and 5B), whereas a fibronectin labelled network and immunoreactive intercellular fibrils were clearly detected in NG97(ht) cells (fig. 5A). In some cells with a known migratory pattern, it was evidenced the accumulation of fibronectin in the lamelipodia cytoplasm extensions. The same pattern was observed for laminin and seems to be more intense in polinucleated cells (fig. 5B). The positive control, the cloned human thymic epithelial cell line [30], presented both intracellular and extracellular fibronectin and laminin expressions (not shown).

### Structural and Numerical Chromosomal Abnormalities

**Figure 6:**
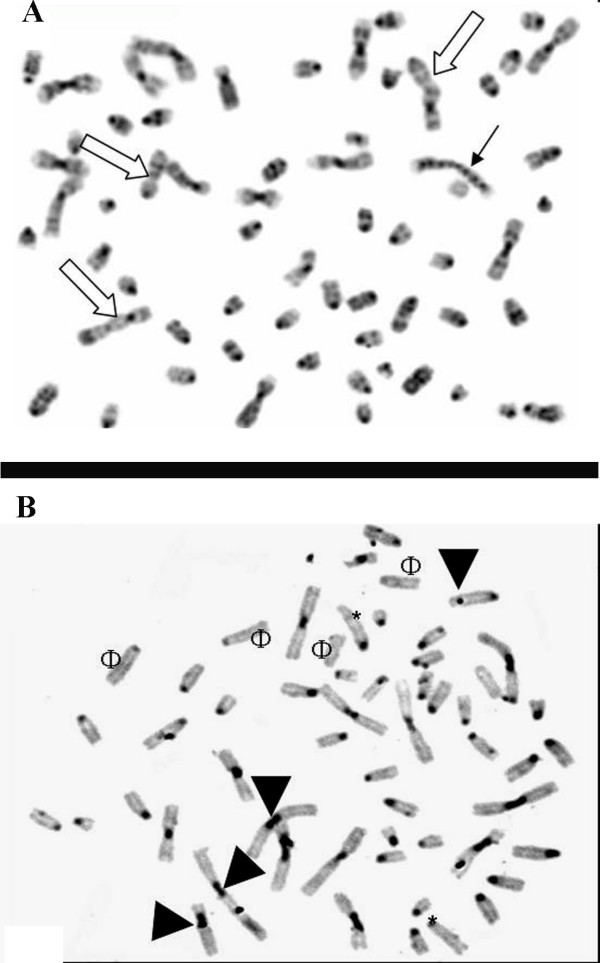
**Digitalizated images from NG97(ht) cells spread metaphases. (A) G-banded chromosomes with 400 bp resolution showing a large amount of chromosomal imbalances represented by figures of bridge (black arrow) and fusioned chromosomes (white arrows).** (B) These breakages formed acrocentric (*) and acentric (Φ) chromosomes which are evidenced by C-band technique. Notice the dicentrics (arrow head) chromosomes and heterochromatin regions.

GTG-band staining applied to the 20^th ^passage of the NG97(ht) cells revealed remarkably fused, translocated segments and acentric fragments (fig. 6A), while several regions of heterochromatin, dicentrics and acrocentric chromosomes were visualized by C-banding (fig. 6B).

**Figure 7:**
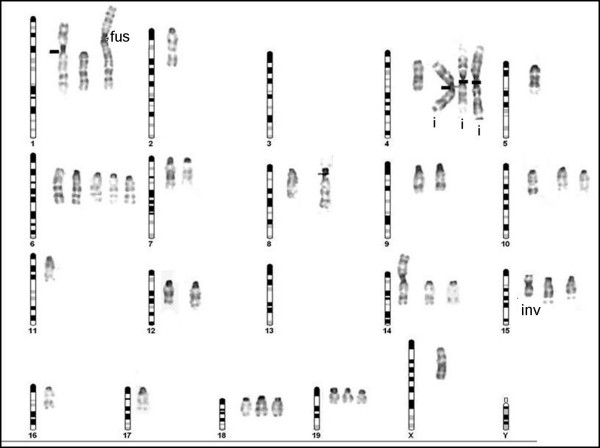
**Mouse representative diagram and the corresponding NG97(ht) murine kayotype.** Note the murine acrocentric chromosomes associated with the cell line composed of isochromosomes (i), fusion (fusion), absences (3 and 13), monossomies (2,5,11,16 and X), trisomies (1,10,14,15,16 and 19), tetrasomies (4 and 6) and chromosomes with pericentric inversion (inv).

The analysis of 100 metaphases showed more than 50 numerical and structural abnormalities, with a modal number of 63 chromosomes. In addition, 89% of metaphases exhibit a range of 20 to 49 acrocentric chromosomes showing a strong correlation with murine banding pattern (fig. 7). The most frequent numerical abnormalities were the monosomies (2, 5, 11, 16, 17 and X), trisomies (1,10,14,15,16 and 19), the tetrasomies (4 and 6), as well as structural alterations like isochromosome formations (murine chr 4 and chr 14), disbalanced fusion (murine: ?→ chr 1) and pericentric inversion (chr 15).

## Discussion

Several studies have associated astrocytomas malignant progression with amplification, over expression or mutation of specific gene sequences [31]. Considering that morphological alterations of the neoplastic tissue are directly linked to these genetic changes, researches have tried to establish which chromosome alterations are directly linked to astrocytomas progression [32,33]. The absence of this correlation is partially due to the fact that astrocytomas present an intrinsic chromosomal instability pattern even in different intra-tumoral regions, disabling this direct correlation.

*In vivo *xenograph tumor models developed by subcutaneous implantation of glioma cell lines in mice are extensively used [6,7] to test therapeutical approaches that target angiogenesis, local invasion and secretion of immune suppressive molecules. These models have the advantages that they are highly tumorigenic, show reproducible growth rates and because their superficial location allows an easily access to tumoral masses. Besides, tumoral mass volume in these dorsal models would mimic the original astrocytoma volume [34] and reproduce the hypothetical "vicious cycle of thrombosis, necrosis, hypoxia, enhanced tumor and metabolic demand" to the development of GBM [35]. Moreover, stable cell cultures derived from these tumors are important tools to therapeutical and biological analysis. However, for immunotherapeutical approaches, the flank microenvironment cells in culture represents a limitation to these models once the inflammatory cytokines and infiltrating cells profile have must be considered for data analysis [36].

### The modulatory pattern of intermediate filaments GFAP and vimentin microarchitectural arrangement in NG97(ht) cells

The astrocytes embryogenic origins can be evaluated by the expression of GFAP, in particular after migration of the progenitor to the radial glia during the CNS development; which is dependent of chemical mediators present in the brain microenvironment [37]. This migratory phase is accompanied by an early vimentin expression, an indicative of the neuroectodermical glial precursor origin. Vimentin establishes a link with microtubules or cytoskeleton filaments to form a dynamic polymerization and depolymerization balance that allows cellular motility [38]. Besides, some glial precursors have a transitory phase named astrocytes type I, where vimentin and GFAP are expressed, which in adulthood originates oligondendrocytes [37]. Thus, the modulatory GFAP and vimentin expression pattern presented by the NG97(ht) cells showed that through cell subcultivation, the reduced immunocytochemistry detection of GFAP is associated to an increased detection of vimentin which may account for the recovery of embryonic characteristics. This feature is observed in other glioma cells [39] and patients diagnosed with astrocytoma grade III and IV [15,16]. Together with GFAP immunodetection decrease and vimentin enhance patterns; there is an increase on cell mitosis concomitant to a decline in cell doubling time. This may be associated with loss of GFAP expression *in vivo*, frequently found in high grade astrocytomas, although this event does not constitute a mandatory step in tumor development and it only represents the cell evolution towards an undifferentiated state. In this sense, some kinases related to cell cycle regulation, like kinases C and cdc2, also may participate of phosphorylation and depolymerization processes of cytoskeleton intermediary filaments at the beginning of cell division. So, the absence of the main component of astrocytes cytoskeleton, GFAP, would increase enzyme accessibility to substrats, resulting in progressive cell cycle acceleration [40]. It is also important to note that the increase of positive vimentin cells cannot be attributed to the possible NG97 cell fusion with the murine stroma in the xenotransplant, because the monoclonal antibodies anti-vimentin clone V9 used in the immunocytochemistry recognizes specifically the human vimentin [41]. However, the monoclonal antibodies anti-vimentin 3B4 clone used in Western Blot reaction recognizes both human and murine proteins. Thus, Ciesielski-Treska and colleagues documented before the ~41,8 kDa vimentin sub band as an artifact resulted from protein extraction [42].

### High migratory and malignant potential driven by the NG97(ht) expression of α5β1, fibronectin and laminin

The increased aggressiveness of astrocytomas is accompanied by the acquisition of cellular migratory potential [18], from the modulation of integrins and their receptors expression with an increase of lamellipodia cytoplasm extensions and diminution of focal adhesion to the extracellular matrix [43].

Integrins are composed of two non covalently associated subunits (α and β) that form a binding pocket for specific sequences or domains in extracellular matrix (ECM) molecules; the most well known is the tripeptide RGD (argenin-glycin-aspartic acid) domain found in fibronectin and laminin ECM molecules. In gliomas, the α4, α5 and α6 molecules combined with β1 subunit in cellular membrane form the integrin adhesion molecules that can also play a role in intracellular cell signaling that leads, at last, to promote an increase in proliferation rates [44].

This integrin molecule heterodimerization and clustering can be modulated by RGD polypeptides concentration [44]. The absence of α4β1 expression observed in NG97(ht) was also documented previously in gliomas cell lines [44]. Besides, some studies only demonstrated the expression of α5β1, α6β1 and their ligands both *in vivo *and *in vitro*. The fibronectin ligand for the α5β1 integrin was described by some authors to be increased or decreased in gliomas influencing its invasive patterns. A high-level expression of α5 monomeric integrin concomitant to the β1 intermediate expression was observed, suggesting an unfunctional α5β1 integrin expression. Indeed, some authors describe this fact as a result of differential interaction of integrin molecules in different tumoral mass regions. Specifically in the tumor core, α5β1 is functionally expressed to modulate cell divisions, while α5β1 interaction with fibronectin in the ECM induces cell migration [45]. It is hypothesized that the reduced β1 integrin and the high expression of α5 would correspond to an increased expression of the Mgat5 enzyme during malignant transformation resulting in an increase of β1,6 glycosides ramifications at the β1 subunit. This process could reduce integrin dimerization and clusterization of β1 and α5 [46], once the fibronectin is synthesized and released to the extracellular compartment of the NG97(ht) cells, this would be a high stimuli to α5β1 expression. In the NG97(ht) cells, no expression of α6β1 integrin was observed, although its laminin ligand was present in the cytoplasm. It is reasonable to ponder that the α6β1 expression absence is a result of a lack of stimuli from the microenvironment once laminins were detected in the NG97(ht) mainly in the cytoplasm. Further experiments aiming to assess the integrins functionalities in the cells NG97(ht) will be developed in our laboratory to elucidate these questions.

### The chromosomal abnormalities as a possible explanation for the spontaneous tumoral progression

Also associated to malignant transformation, chromosomal alterations in glioma lineages are poorly described in the literature, while many cytogenetical evaluations in patients were reported. Nevertheless, aneuploidy, hyperploidy or numerical abnormalities documentations presented similarities between literature data in gliomas, pointing to a correlation with the tumoral phenotype [47]. Accordingly to some authors, the aneuploid phenotype would not be the cause, but the consequence of cumulative alterations provided by the tumoral microenvironment [22,26]. The chromosomal instability caused by cytogenetical heterogeneity is responsible for the malignant progression but is still to be fully elucidated [47]. Jacobsen and colleagues [25] demonstrated that mice inoculated with human-derived breast cancer cell lines (BC6) presented cell fusion with murine stroma (BJ3Z), leading to an increased aggressiveness. Pawlek [24], in his review on melanomas, showed solid evidences that stroma immune system cells from tumoral host fusioned with tumoral cells, not only causing aneuploidy but contributing for other mechanisms related to the malignant phenotype including aberrant glycosylation and metastasis. The NG97(ht) karyotyping and immunophenotyping are in agreement with the cell fusion phenomena, once murine chromosomes were observed in the NG97(ht) cells as well CD86 and CD44 murine proteins detected by flow cytometry (data not shown). Also, a possible increase of aggressiveness, given by the reduction of the doubling time from 72 h at NG97 cell establishment [8] to currently 25 h was noticed to occur within the NG97(ht) cells. Nevertheless, the evaluation of these murine chromosomes translocated fragments with human chromosomes or *vice *and *versa *by concomitant human/murine Spectral Karyotyping, as well as the possible chimerisms, must be an objective of future studies. In addition, the evaluation of human cryptic chromosomes analysis by Fluorescent Hybridization could be an important target for further researches.

## Conclusion

The NG97(ht) cells possesses embryonic recovery patterns of intermediate filaments and adhesion molecules expression that corroborate the astrocytoma grade IV diagnosis after NG97 inoculation in nude mice. Furthermore, the chromosomal imbalances and a tumoral cell fusion with the murine stroma cells in the xenotransplant give clues to the spontaneous *in vitro *cell progression to the most aggressive astrocytoma phenotype. However, further studies are necessary to address unanswered questions regarding the *in vitro *tumoral progression of the NG97(ht) cells, aiming to translate these events to the clinical practice and management of astrocytoma tumors.

## Competing interests

The authors declare that they have no competing interests.

## Authors' contributions

This work is part of a Doctor's Dissertation by CMLM. All authors intensely discussed these results.

## Pre-publication history

The pre-publication history for this paper can be accessed here:


